# Lithium-Induced Hypercalcemia Presenting as Catatonia in a Patient With Schizoaffective Disorder

**DOI:** 10.7759/cureus.44114

**Published:** 2023-08-25

**Authors:** Satesh Seegobin, Shantale Williams, Muzammil Hyder, Sachidanand Peteru

**Affiliations:** 1 Psychiatry, Ross University School of Medicine, Bridgetown, BRB; 2 Psychiatry, Jamaica Hospital Medical Center, Queens, USA; 3 Consultation-Liaison Psychiatry, Flushing Hospital Medical Center, New York, USA

**Keywords:** calcium, toxicity, catatonic, parathyroid, hyperparathyroidism, schizoaffective disorder, lithium, hypercalcemia, catatonia

## Abstract

Catatonia, a relatively rare and less explored consequence of lithium-induced hypercalcemia, represents a notable yet understudied side effect. Lithium is utilized in the management of acute mania and as a maintenance therapy for bipolar disorder. However, the specific catatonic presentation resulting from hypercalcemia remains poorly understood. Here, we present a case study involving a 55-year-old male with a history of schizoaffective disorder, bipolar type, who had been receiving lithium therapy. The patient presented with catatonia and altered mental status. Manifesting as mutism, rigidity, immobility, and staring, these symptoms were subsequently attributed to hyperparathyroidism-induced hypercalcemia. Markedly elevated levels of both calcium and parathyroid hormone (PTH) were detected in the patient's laboratory results. The patient's lithium therapy was promptly discontinued. Serum calcium and PTH levels began to decrease gradually and returned to normal limits over 29 days. The patient returned to his baseline level of functionality. There was a notable improvement in his mental status and his ability to communicate using simple sentences. This case underscores the significance of recognizing uncommon clinical presentations of hypercalcemia in patients undergoing chronic lithium therapy. Given the broad range of neuropsychiatric manifestations associated with hypercalcemia, it is crucial to enhance our understanding of this phenomenon and develop the capacity to differentiate it from primary psychiatric disturbances.

## Introduction

The primary objective of this case report is to present an intriguing clinical scenario involving a patient exhibiting an unusual manifestation of catatonia as a result of lithium-induced hypercalcemia. Lithium, a commonly employed medication for the management of acute mania and maintenance treatment of bipolar disorder (BPD), has been extensively associated with well-documented adverse effects such as chronic kidney disease (CKD) and hypothyroidism. However, emerging research is shedding light on a lesser-known complication of lithium therapy, namely, hypercalcemia [1]. It is postulated that lithium exerts its influence on calcium homeostasis through the disruption of calcium-sensing receptors (CaSR) located on the parathyroid gland, consequently elevating the set point of parathyroid function [2]. Consequently, parathyroid hormone (PTH) secretion increases, resulting in a subsequent rise in serum calcium levels. Nevertheless, cases of lithium-induced hypercalcemia have been reported with normal PTH levels, indicating the potential involvement of alternate mechanisms [3].

Hypercalcemia is well recognized for its ability to precipitate neuropsychiatric symptoms, such as delirium and depression, alongside somatic manifestations, including constipation and bone pain [4]. Catatonia, an infrequent but plausible expression of these neuropsychiatric effects, has been associated with hypercalcemia [5]. Given the diverse range of consequences stemming from lithium-induced hypercalcemia, it is imperative to differentiate them from primary psychiatric disorders [6]. In this context, we present a case of a 55-year-old male with a previous psychiatric history of schizoaffective disorder, bipolar type, and ongoing lithium therapy. The patient presented with catatonia and altered mental status, necessitating a thorough examination and evaluation to discern the underlying etiology.

## Case presentation

A 55-year-old Caucasian male with a past psychiatric history of schizoaffective disorder, bipolar type, maintained on long-term maintenance therapy with lithium carbonate presented to the emergency room with confusion, mutism, inhibited movement, staring, and posturing. According to his relatives, the patient was diagnosed with schizoaffective disorder in his late 30s after presenting with a depressive episode. He continued to present with mainly depressive episodes since then. The patient was placed on lithium 300 mg three times a day (TID) a few years prior to this hospitalization by his psychiatrist with good effect. On admission to the hospital, the patient presented with auditory hallucinations, generalized confusion, and mutism. CT imaging without contrast of the patient's brain was unremarkable.

Lithium levels were obtained, which showed a level of 0.6 (normal: 0.6-1.2). Serum calcium level was above normal limits, with a level of 12.0 mg/dL (normal: 8.4-10.2mg/dL), and serum PTH level was greater than two times the normal value of 135.5 pg/mL (normal: 10-65 pg/mL). Brain CT without contrast was normal. Bone density was normal. Calcitonin, thyroid-stimulating hormone, and creatinine were within normal limits.

The patient was administered Ativan 2 mg oral (PO) and IV normal saline solution for hydration. Lithium therapy was discontinued. Five days after admission, serum calcium level decreased from 12.0 mg/dL to 9.5 mg/dL and psychiatric symptoms improved. The patient no longer exhibited mutism, rigidity, and staring. He responded to simple commands and began using two to three-word sentences. PTH levels were monitored on days one, seven, 14, and 28, and then one month later (30 days after day 28). PTH levels decreased from 135.5 pg/mL to 64.0 pg/mL in 58 days (Table 1) displaying a gradual stabilization of PTH levels over time. These data demonstrate that long-term lithium therapy can cause a hyperparathyroid-induced hypercalcemic state, leading to a primary psychiatric manifestation of catatonia.

**Table 1:** Serum parathyroid hormone and calcium levels over time

Day	Parathyroid hormone (pg/mL) (normal = 10-65 pg/mL)	Calcium (mg/dL) (normal = 8.4-10.2 mg/dL)
1	135.5	12
7	112	-
14	98.5	-
28	87	-
58	64	9.5

## Discussion

We present a case showcasing the development of catatonia secondary to lithium-induced hypercalcemia in a patient with long-standing treatment-resistant schizoaffective disorder. This report contributes to the existing literature emphasizing the potential serious medication effects associated with lithium treatment, which can complicate the course of psychiatric illness.

A recent study examining 60 publications demonstrated that approximately 10% of lithium-treated patients had an absolute risk for hypercalcemia and hyperparathyroidism compared to unexposed individuals. However, the true prevalence of lithium-induced hypercalcemia and hyperparathyroidism remains unknown due to the lack of population-based data [7].

Lithium exerts its hypercalcemic effects through several mechanisms. It enhances renal calcium reabsorption in the loop of Henle and can independently stimulate the release of PTH. By antagonizing CaSR, lithium alters the set point for PTH secretion, resulting in an elevated threshold of serum calcium concentration required to suppress PTH output by the parathyroid gland. Consequently, excessive PTH secretion occurs, as the parathyroid cells perceive a decrease in extracellular calcium concentration [8,9]. Chronic stimulation or unmasking of underlying subclinical pathology can also contribute to lithium-induced alterations in parathyroid gland activity and structure [10,11].

Patients receiving lithium for more than three years may exhibit a three-fold increase in parathyroid mass compared to those on lithium for a shorter duration [10]. These hyperplastic changes in the parathyroid gland can lead to a clinical presentation resembling primary hyperparathyroidism, referred to as "lithium-associated primary hyperparathyroidism" - a condition that often takes years to develop [12]. In cases where surgery is not feasible, cinacalcet is the first-line treatment for lithium-associated primary hyperparathyroidism [13]. Notably, the patient demonstrated complete resolution of laboratory measures with a relatively lower dose of cinacalcet (30 mg twice per day), typically prescribed for managing secondary hyperparathyroidism, as opposed to primary hyperparathyroidism.

Catatonia presented another challenging aspect of the patient's clinical course. A meta-analytic study involving 73 studies indicated an overall pooled prevalence of catatonia of 9.0% among 110,774 individuals and 20.6% in the presence of medical and neurological comorbidities [14]. The majority of these studies focused on psychiatric inpatients. In this specific patient population, catatonia exhibited comparable prevalence rates in mood disorders (as seen in major depressive or bipolar catatonia) and schizophrenia but was more prevalent in bipolar disorder compared to schizophrenia or unipolar major depressive disorder [15]. Schizophrenia spectrum and other psychotic disorders make up to 25% of consultation requests to consultation-liaison psychiatry services in hospitals and it is important to be aware of the potential complications of lithium treatment in these populations [16]. While the resolution of catatonia appeared to coincide with the normalization of calcium levels, it is important to note that the elevated PTH in this patient served as a marker of excessive parathyroid gland activity and exerted its effect through alterations in calcium levels, without directly affecting mental status. It is important to note that lithium-induced electrolyte imbalances can complicate a variety of treatments, therapies, and procedures, including electroconvulsive therapy [15].

## Conclusions

In conclusion, this case report highlights the occurrence of catatonia as a rare manifestation of lithium-induced hypercalcemia in a patient with long-standing treatment-resistant schizoaffective disorder. The development of hypercalcemia and subsequent PTH dysregulation in response to lithium therapy underscores the importance of monitoring calcium and PTH levels in individuals receiving long-term lithium treatment. The mechanisms by which lithium induces hypercalcemia involve alterations in renal calcium reabsorption and disruption of calcium-sensing receptors, leading to excessive PTH secretion. These changes can result in the development of hyperplasia in the parathyroid gland, mimicking primary hyperparathyroidism.

Management of lithium-induced hypercalcemia may require discontinuing lithium therapy and considering alternative treatment options, such as cinacalcet, particularly when surgical intervention is not feasible. While the resolution of catatonic symptoms coincided with the normalization of calcium levels, it is important to recognize that the elevated PTH levels primarily reflect parathyroid gland activity and do not directly impact mental status. This case report emphasizes the need for healthcare professionals to remain vigilant for the potential psychiatric and medical complications associated with lithium therapy. Early recognition and appropriate management are crucial in minimizing the adverse effects of lithium treatment and optimizing patient outcomes. Further research is warranted to elucidate the underlying mechanisms of lithium-induced hypercalcemia and to develop strategies for early detection and prevention of such complications.
